# Hemodynamics in the treatment of pseudoaneurysm caused by extreme constriction of aortic arch with coated stent

**DOI:** 10.3389/fcvm.2024.1363230

**Published:** 2024-08-20

**Authors:** Lanlan Li, Yiwei Wang, Ping Jin, Tingting Yang, Guangyu Zhu, Yuxi Li, Jiayou Tang, Yang Liu, Jian Yang

**Affiliations:** ^1^ Department of Cardiovascular Surgery, Xijing Hospital, Air Force Medical University, Xi’an, Shaanxi, China; ^2^ School of Energy and Power Engineering, Xi’an Jiaotong University, Xi’an, Shaanxi, China; ^3^ Department of Ultrasound Medicine, Xijing Hospital, Air Force Medical University, Xi’an, Shaanxi, China

**Keywords:** coarctation of aorta, aortic pseudoaneurysm, hemodynamics, balloon-expandable stent, surgical strategy

## Abstract

**Objectives:**

To evaluate the changes in distal vascular morphology and hemodynamics in patients with extremely severe aortic coarctation (CoA) after covered palliative (CP) stent dilation with different surgical strategies.

**Materials and methods:**

Perioperative computed tomography angiography and digital subtraction angiography were utilized to construct three aortic models with varying stenosis rates and one follow-up model in a patient with extremely severe CoA. The models included: an idealized non-stenosed model (A: 0%), a model post initial stent deployment (B: 28%), a model post balloon expansion (C: 39%), and a model 18 months after post-balloon expansion (D: 39%). Consistent boundary conditions were applied to all models, and hemodynamic simulation was conducted using the pure fluid method.

**Results:**

The narrowest and distal diameter of the stent increased by 34.71% and 59.29%, respectively, from model B to C. Additionally, the distal diameter of the stent increased by −13.80% and +43.68% compared to the descending aorta diameter, respectively. Furthermore, the ellipticity of the maximum cross-section of the aneurysm region in model A to D continued to increase. The oscillatory shear index at the stenosis to the region of the aneurysm were found to be higher in Models A and B, and lower in Models C and D. At the moment of maximum flow velocity, the blood flow distribution in models A and B was more uniform in the widest section of the blood vessels at the distal end of the stenosis, whereas models C and D exhibited disturbed blood flow with more than 2 eddy currents. The time-averaged wall shear stress (TAWSS) decreased in the distal and basal aneurysms, while it significantly increased at the step position. The aneurysmal region exhibited an endothelial cell activation potential value lower than 0.4 Pa^−1^.

**Conclusion:**

In patients with extremely severe CoA, it is crucial to ensure that the expanded diameter at both ends of the CP stent does not exceed the native vascular diameter during deployment. Our simulation results demonstrate that overdilation leads to a decrease in the TAWSS above the injured vessel, creating an abnormal hemodynamic environment that may contribute to the development and enlargement of false aneurysms in the early postoperative period.

**Clinical Trial Registration:**

ClinicalTrials.gov, (NCT02917980).

## Introduction

Extremely narrowed aortic arch is a rare and severe congenital heart disease in which patients often present with upper extremity hypertension, making activities limited, and stenting of the aortic arch is currently the modality of choice for the treatment of coarctation of the aorta (CoA) (1–3). When employing Cheatham Platinum (CP) stents for CoA treatment, the balloon-in-balloon (BIB) catheter is typically not inflated to the extent that it fully matches the size of the stenotic region within the vessel, which makes the mid-section of the stent present a waist sign in the postoperative period, and the stent has poor morphology and a high rate of shortening after overexpansion, which can damage the endothelium and cause iatrogenic pseudoaneurysm in the distant future of the procedure (4, 5). Therefore, how to find a more appropriate surgical strategy for patients with extreme narrowing of the aortic arch is a major clinical problem that needs to be explored and solved under the constraints of guaranteeing the safety of the procedure, the long-term effectiveness of the postoperative period, and the feasibility of the surgical program in line with the actual operation of the clinic (6–9).

It has been observed that diverse hemodynamic conditions can influence the morphological development of blood vessels. In the presence of hemodynamic irregularities within the vessel, there is a risk of aneurysm, aortic dissection, and atherosclerosis (6, 7). When the vessel undergoes an interventional procedure, the hemodynamic environment is significantly altered (7, 8). Evaluating the efficacy of stent implantation in the clinical setting involves assessing changes in pressure and flow velocity immediately post-implantation. Over the long term, the adaptation of vessels to these changes and the potential future complications can be predicted by analyzing alterations in vascular forces and blood flow patterns (8, 9). These biomechanical parameters can be determined using Computational Fluid Dynamics (CFD) numerical simulation techniques.

This study involved a retrospective analysis of a patient who had undergone CoA stenting and subsequently developed a pseudoaneurysm. Numerical simulations, utilizing multimodal images of the patient, were conducted to compare the changes in hemodynamic parameters between the anticipated ideal state and the actual intraoperative conditions post-procedure (6–8). The objective is to predict the risk factors of complications, such as pseudoaneurysm, after the treatment of extreme CoA using morphological and biomechanical analysis (9, 10). Additionally, this research methodology can aid in identifying a more suitable procedure strategy for patients with extreme aortic arch narrowing, considering both surgical safety and long-term effectiveness, while ensuring practical feasibility within clinical practice (11, 12). This will serve as a robust foundation for the future development and implementation of stent implantation strategies for aortic arch narrowing.

## Materials and methods

### Baseline characteristics

A male patient presenting with a narrowed aortic arch and intractable hypertension (upper extremity blood pressure of 210/110 mmHg) was selected for this study. The patient had a dissected stenosis (dissected length of 6 mm) and preoperative measurements indicating a left radial artery blood pressure of 185/110 mmHg, right femoral artery blood pressure of 75/60 mmHg, and an stenosis pressure difference of 110 mmHg.

### Procedures

Intraoperatively, DSA-guided transcatheter stenting was performed using 39-mm-length overlay CP stents, along with a 20 mm Numed BiB balloon dilatation. During the procedure, the stent underwent initial balloon expansion, and angiography revealed the presence of stenosis, with the stent being well adhered to the vessel wall. Monitoring of the blood pressure using a pigtail catheter positioned at the proximal and distal ends of the stenosis yielded a maximum systolic pressure difference of 13 mm Hg. Due to the apparent stenosis and the pressure difference not meeting the standard range, the surgeon applied high pressure to the balloon for a second time to expand the stent, resulting in a reduced pressure difference of 4 mmHg. Consequently, the patient's hypertension was alleviated, and the procedure was successfully concluded.

### Computed tomography angiography (CTA) images

The CTA images were acquired using a Siemens dual-source Flash CT, covering the scanning range from the skull base to the bilateral groin, with a slice thickness of 1 mm and slice spacing of 0.8 mm. The scanning direction was head to foot, with a tube voltage of 100 KV and an automatically adjusted tube current ranging from 200 mA to 600 mA. A contrast agent triphasic injection scheme was implemented as follows: I. 350 mgI 70–80 ml at a flow rate of 4–5 ml/s, II. 350 mgI 20 ml at a flow rate of 1.5 ml/s, III. saline 30–40 ml at a flow rate of 4–5 ml/s.

### Geometric modeling

**Figure 1:**
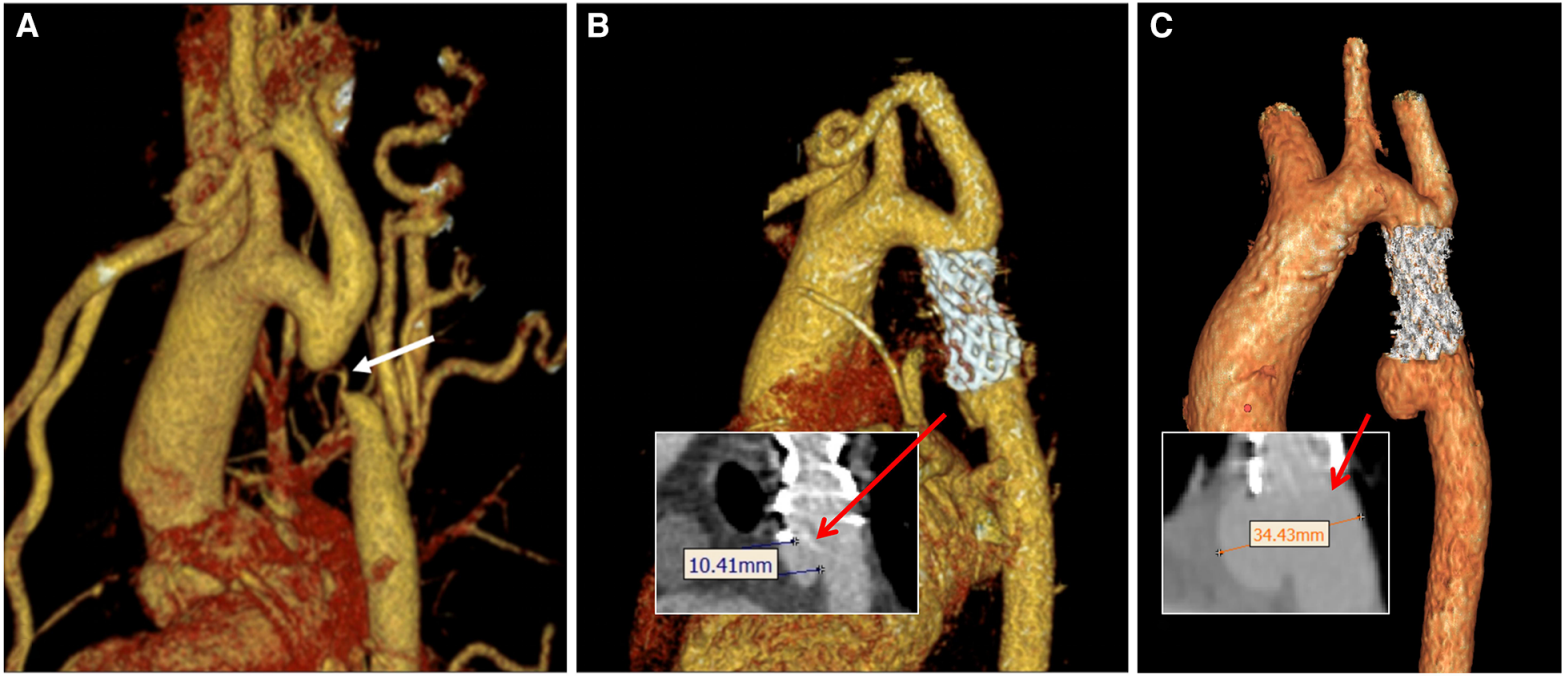
Three-dimensional images of the aorta during perioperative CTA in patient. **(A)** Preoperative image; **(B)** postoperative-3 days; **(C)** postoperative-18 months. The white arrow indicates the position of the stenosis. The red arrows indicate the neck of the aneurysm in B and the distance between the aneurysm dome and the parent artery in C, respectively.

**Figure 2:**
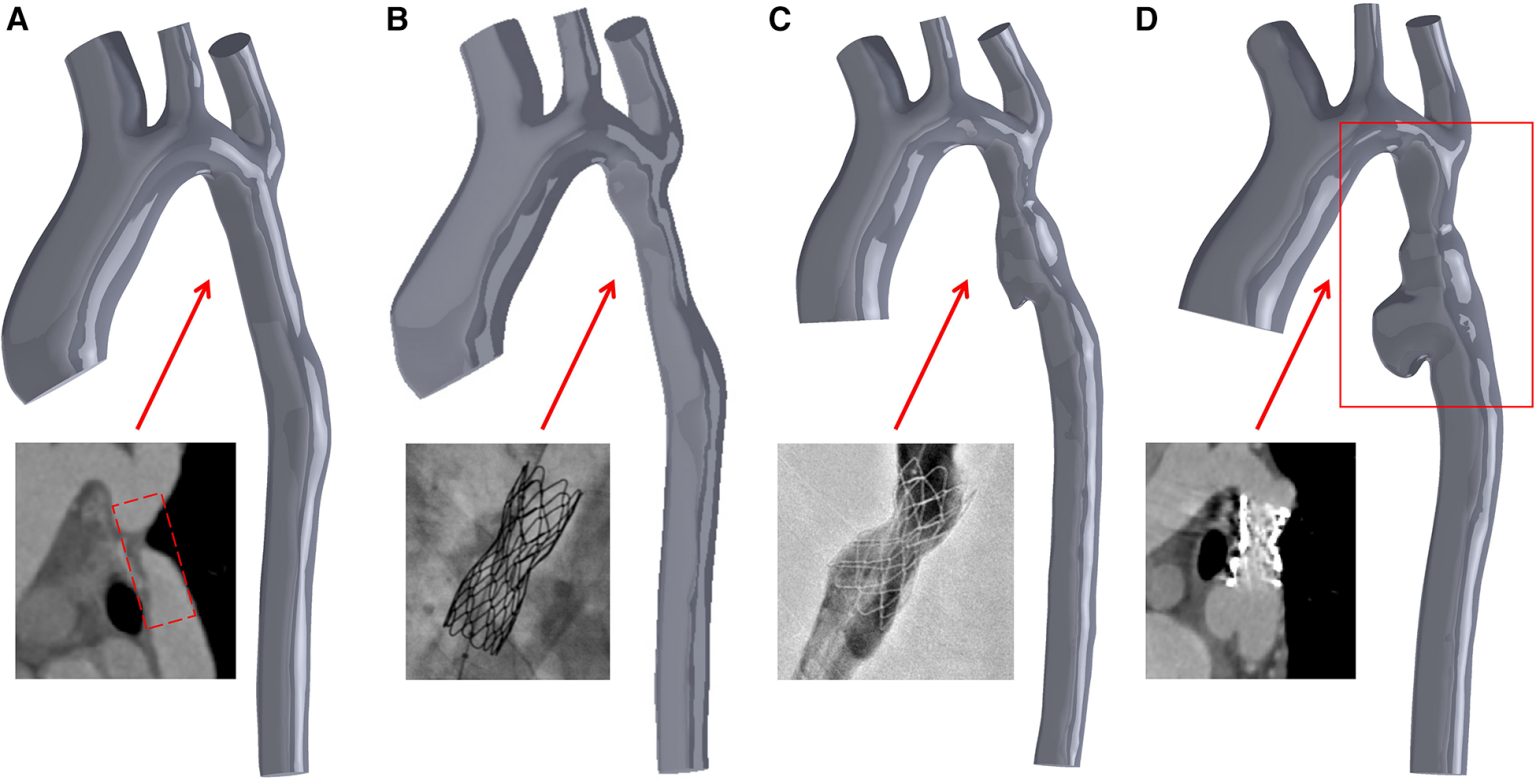
Three-dimensional digital models were constructed based on CTA and DSA images. Model A represents the idealized expansion of the stent to match the vessel diameter; model B signifies the initial partial expansion of the stent; model C portrays the subsequent over-expansion of the stent; and model D showcases the vessel model 18 months after the second over-expansion of the stent. Specifically, model C corresponds to a 3-day postoperative model depicted in Figure 1B, while model D corresponds to the 3D CTA images shown in Figure 1C.

In this study, we acquired computed tomography angiography (CTA) images of the patient, encompassing preoperative CTA, CTA at 3 days postoperatively, and CTA at 18 months postoperatively (Figure 1). The Mimics Innovation Suite 21.0 software was utilized to interpret the patient's CTA in DICOM format. First, we developed model A, aimed at achieving perfect correction, by utilizing preoperative CTA images to replicate the stenosis location with the same diameter as the descending aorta, demonstrating a stenosis rate of 0.0%. Subsequently, model B was constructed for optimal intervention, which entailed measuring the stent height and diameters at both ends of the stent based on the digital subtraction angiography (DSA) images following the initial intraoperative balloon dilatation and comparing these measurements with those from the preoperative CTA images. After realizing that the DSA images were not scaled, the stent shape and size at different angles following the initial surgical intervention were obtained based on the intraoperative dynamic DSA images. Subsequently, Model B, with a stenosis rate of 28%, was virtually constructed using the preoperative CTA images. Subsequently, Model C was developed following the second intervention, signifying overcorrection and the conclusion of the procedure after the second balloon dilatation. Model C, representing a stenosis rate of 39% in the actual state, was constructed based on the review of CTA images at 3 days postoperatively. Lastly, a model was constructed for the patient at 18 months after the second dilatation of the stent, and a model of the patient at 18 months after the second dilatation of the stent was constructed by using the review CTA images to directly construct model D, with a stenosis rate of 39%. Model D was utilized to validate the findings of Model C and to monitor the progression of aneurysm development. It is noteworthy that the model was simplified by deleting the distal collateral vessels in the stenosis of the descending aorta (Figure 2).

### Numerical simulation methods

**Figure 3:**
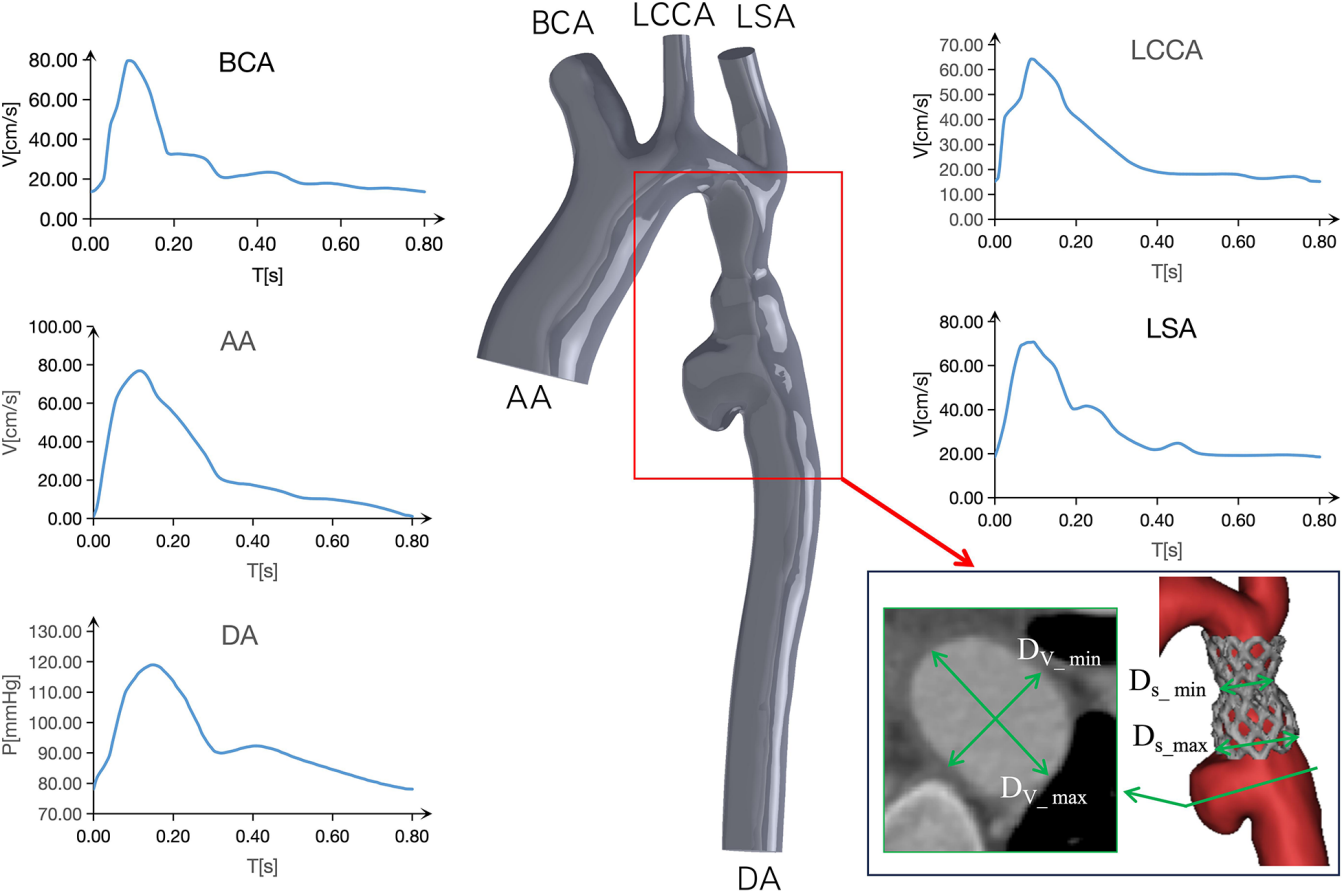
Boundary conditions of models and schematic representation of the plane of correlation analysis.

The blood flow was modeled as an incompressible Newtonian fluid with laminar characteristics. Key parameters included a blood density of 1,150 kg/m^3^, blood viscosity of 0.0035 Pa·s, and Poisson's ratio of 0.45. Both the vessel wall and stent position were treated as rigid walls with no slippage. Pure fluid analysis was conducted using the CFX module in Ansys 2021 R1 software, with a boundary layer of 8. Additionally, tetrahedral meshers were created in the fluid domain using Ansys ICEM CFD (10, 11). As depicted in Figure 3, boundary conditions were established based on postoperative ultrasound and monitoring, enabling the determination of flow velocity at the center point of the ascending aorta (AA), brachiocephalic trunk (BCA), left common carotid artery (LCCA), and left subclavian artery (LSA) (13). At the same time, a 5F pigtail catheter with a pressure sensor was inserted into the distal end of the descending aorta (DA) through the femoral artery. The pressure waveform of the outlet DA and the heart rate (75 beats/min, corresponding to a cardiac cycle of 0.8 s) were recorded using an ECG monitor (14). The transient simulations demonstrated that the model achieved convergence in calculating the results for three cardiac cycles. As a result, the findings of the fourth cycle were extracted for analysis in this study. (The measurement data for all models, including boundary, stenosis position, aneurysm diameter, stenosis rate, ellipticity, etc., are presented in Table 1. The corresponding measurement positions for each parameter are illustrated in Figure 3.)

**Table 1:** The diameters of aortic at baseline and follow-up.

Model	A (control)	B (1st-expansion)	C (2nd-expansion)	D (Postoperative-18 months)
D__AA_(mm)	27.94	27.73	28.41	30.81
D__BCA_(mm)	19.51	19.46	16.39	15.47
D__LCCA_(mm)	10.02	10.03	8.39	7.37
D__LSA_(mm)	13.37	13.48	11.82	10.28
D__DA_(mm)	14.77	14.85	14.19	16.36
D_S_min_(mm)	18.32	9.22	12.42	11.94
D_S_(mm)	18.32	12.80	20.39	19.67
Percentage of stent stenosis	0%	28%	39%	39%
D_V_max_(mm)	18.45	18.78	23.73	34.43
D_V_min_(mm)	18.30	18.30	18.53	22.76
Ellipticity	1.01	1.03	1.28	1.51

### The post-processing data extraction

The post-processing software CFD-Post 2021 R1 was utilized to calculate essential parameters such as Time-Averaged Wall Shear Stress (TAWSS), Oscillatory Shear Index (OSI), Endothelial Cell Activation Potential (ECAP) (12), velocity, and others. These parameters were displayed using contours, vector diagrams, and various other visualization methods. The pertinent mechanical parameter are provided in Equations (1), (2), and (3), respectively:

(1) $$\mathrm{TAWSS}=\frac{1}{T}\overset{T}{\underset{0}{\int }}|WSS|\mathrm{dt}$$

(2) $$\mathrm{OSI}=\frac{1}{2}\left(1-\frac{\left|\frac{1}{T}\int_{0}^{T}WSS\mathrm{dt}\right|}{\frac{1}{T}\int_{0}^{T}|WSS|\mathrm{dt}}\right)$$

(3) $$\mathrm{ECAP}=\frac{\mathrm{OSI}}{\mathrm{TAWSS}}$$

Wall Shear Stress (WSS) refers to the tangential force exerted on a vessel due to pulsatile blood flow. In formulas (1) and (2), **WSS** represents the instantaneous WSS vector, and T denotes the integration period, specifically referring to a cardiac cycle. TAWSS denotes the average integral value of WSS during a cardiac cycle. Inadequately low WSS can lead to intimal inflammation and platelet deposition, while excessively high WSS can damage the blood vessel wall. OSI is utilized to assess WSS volatility, primarily measuring the degree of WSS direction change during the cardiac cycle. The value ranges from 0 to 0.5, where 0 indicates stable WSS direction throughout the cardiac cycle, and 0.5 indicates significant changes in WSS direction, representing purely oscillatory flow. ECAP serves to characterize the potential for local mural thrombosis in aneurysms. A higher ECAP suggests a greater likelihood of thrombosis, and a value of 1.4 Pa-1 is generally considered critical (15). Therefore, these hemodynamic parameters can be employed to analyze the development of vascular aneurysms and associated complications (16).

## Results

### Morphological feature

Initially, anatomical measurements indicated a progressive decrease in the diameters of the three branch vessels of the aortic arch post-surgery. Notably, at the 18-month mark, the BCA, LCCA, and LSA exhibited reductions of 20.71%, 26.45%, and 23.11%, respectively, compared to their preoperative measurements. In contrast, the diameters of the aortic AA and descending aortic DA increased by 10.27% and 10.77%, respectively, during the same period.

From model B to model C during the two intraoperative interventions, it can be seen that the diameter of the narrowest stenosis position of CoA patients increased from 9.22 mm to 12.42 mm, that is, the diameter of the stenosis position of the stent increased by 34.71% after expansion. The diameter of the distal stent at the stenosis site expanded from 12.80 mm to 20.39 mm, an increase of 59.29%. However, for CP stents, the stenosis rate at the CoA lesion site increased from 28% to 39%. Meanwhile, relative to the diameter of the descending aorta, the diameter of the distal end of the stent decreased by 13.80% in model B and increased by 43.68% in model C (Table 1).

Figures 1, 2 show that following the second intervention, an asymmetrical “step” pattern emerged on the inner curvature of the aorta, located 10.41 mm from the distal end of the stent. The angle formed at this position became increasingly sharper over the 18 months after surgery. The DSA image reveals a deepening of the angiographic development at the top of the “step”. According to the two follow-up examinations, the vascular wall in this region continues to locally expand, leading to the formation of aneurysms. The maximum aneurysm diameter (DV_max), measured as the distance between the aneurysm dome and the parent artery, gradually increases from Model A to Model D, with growth rates of 1.79%, 26.36%, and 45.09%, respectively. The narrowest diameter (DV_min) grew more slowly, with growth rates of 0%, 1.26%, and 22.83%. Concurrently, the ellipticity of the blood vessels at this cross-section increased by 1.98%, 24.27%, and 17.97% across the models.

### Hemodynamic characteristics

**Figure 4:**
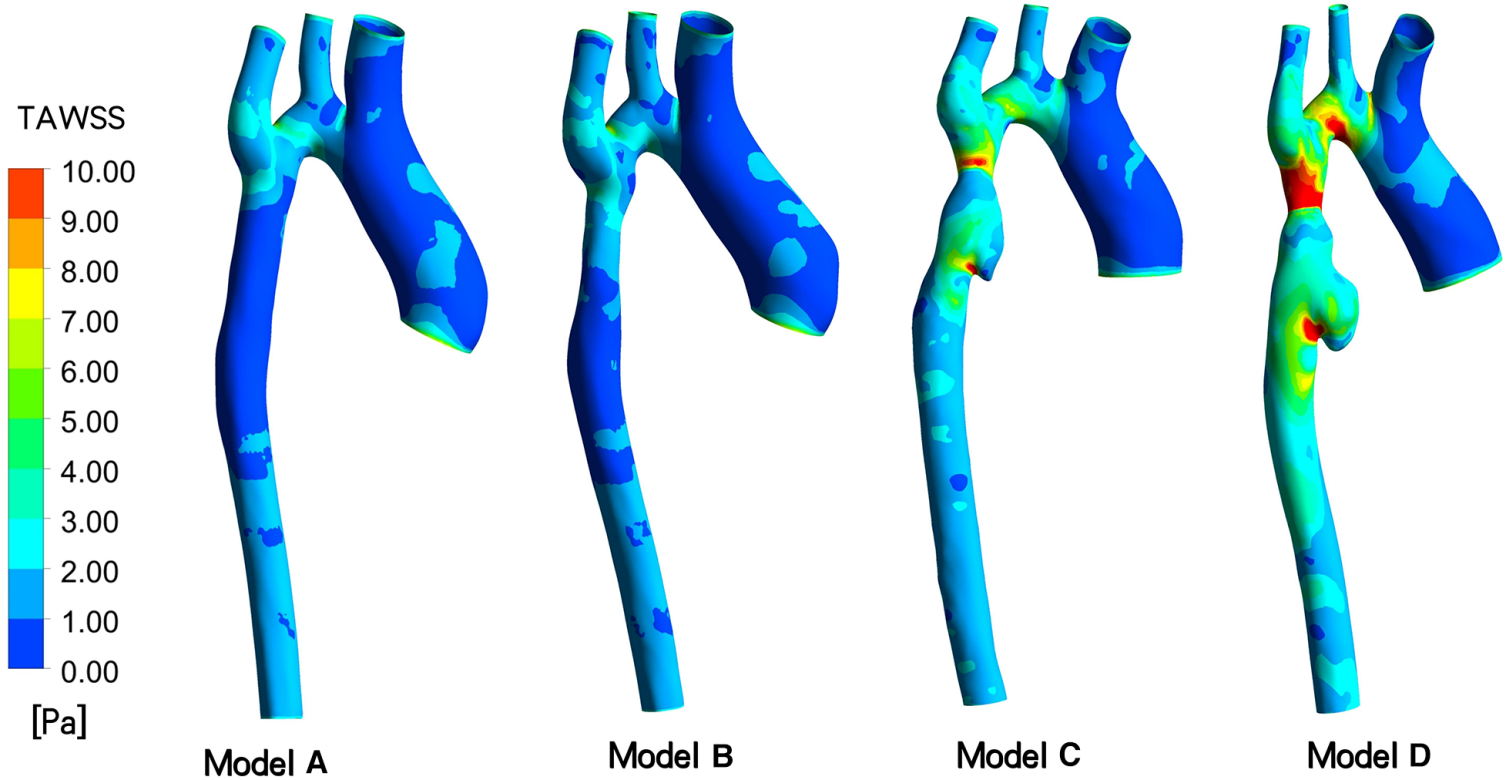
Depicts time-averaged wall shear stress (TAWSS) contour of models A to D indicated in different follow-up.

TAWSS: As depicted in Figure 4, the TAWSS of the vascular wall in the aneurysm region in models C and D, reviewed after surgery, was higher compared to the ideal Model A and Model B, which exhibited insufficient stent expansion during the initial intervention. In model C, the TAWSS on both sides of the short axis of the aneurysm fell within the range of normal vascular stress, while the local TAWSS at this position in Model D was in the 5∼6 Pa range, slightly higher than the normal value. At 18 months post-surgery, the TAWSS at the distal end of the long axis of the aneurysm and at the lowest end of the aneurysm in Model C was lower than 1 Pa, whereas in Model D, it was within the normal range of 1∼4 Pa. With the growth of the aneurysm, the TAWSS at the “step” position in Model D increased significantly, with an area of more than 5 Pa expanding. Furthermore, in Model C, characterized by excessive stent expansion during the second intervention, a step-type aneurysm structure emerged in the blood vessels at the distal end of the stent, leading to an overall increase in TAWSS at this position, particularly at the step-type location.

**Figure 5:**
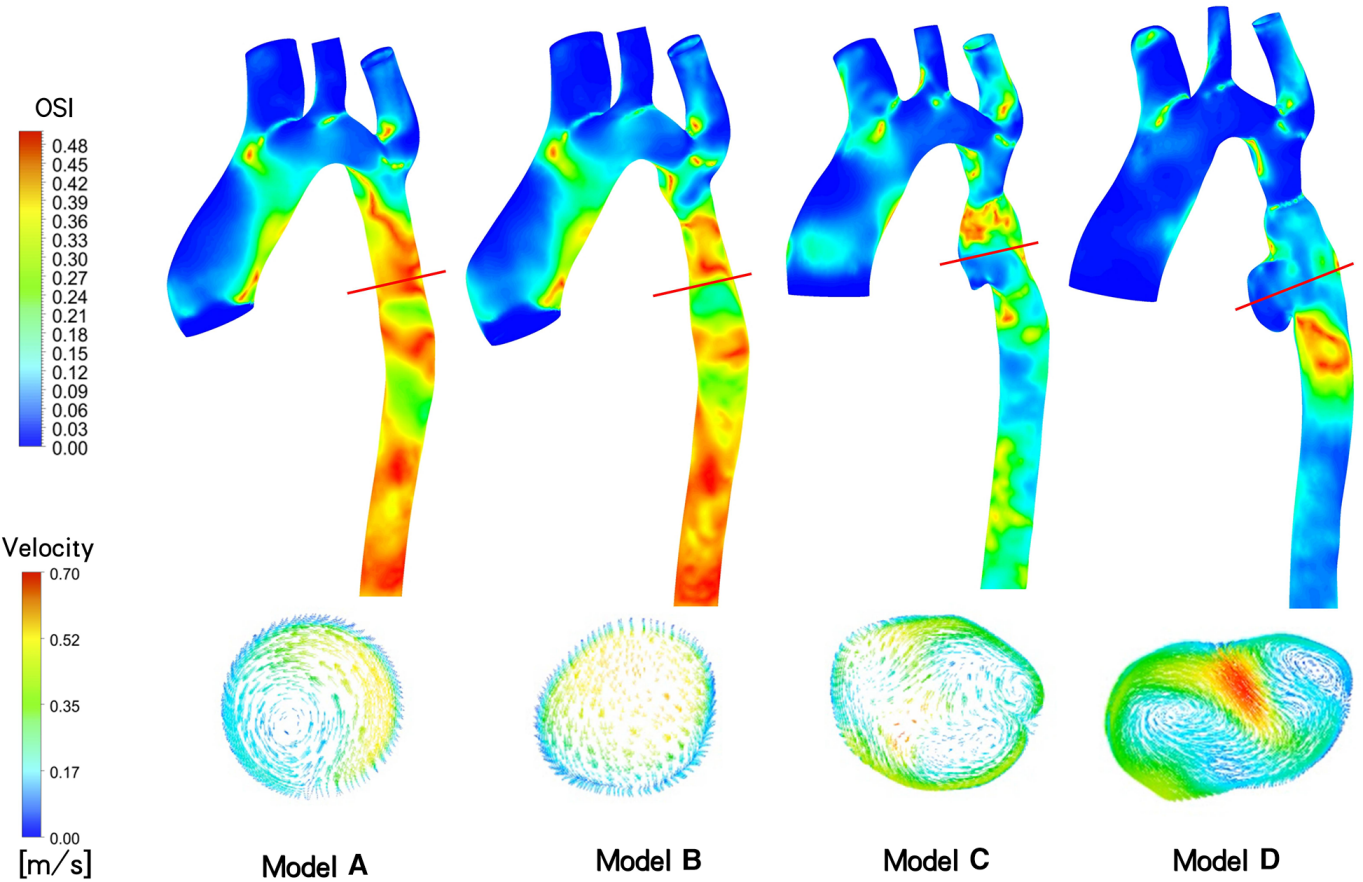
Aortic Oscillatory Shear Index (OSI) contour and cross-sectional velocity vectors at the site of aneurysm generation of maximum flow rate at different follow-up time (*t* = 0.096s).

OSI and Velocity: In Figure 5, following the initial intervention (Model B), the OSI value at the distal stent closely approximates that of an idealized normal individual (Model A), ranging from 0.30 to 0.45. However, at 3 days (Model C) and 18 months (Model D) after overdilation, the blood vessels downstream of the stent continue to dilate. Consequently, the OSI at the aneurysm gradually decreased. in model C, the OSI is primarily concentrated at 0.06–0.12, and in model D, it ranges from 0.00 to 0.09. The maximum inlet flow velocity occurs at t = 0.096 s during one cardiac cycle. The velocity distribution appears more uniform in model A and B, model C shows mild blood flow disturbance, and in model D, up to three instances of rotational flow are observed in this cross-section.

**Figure 6:**
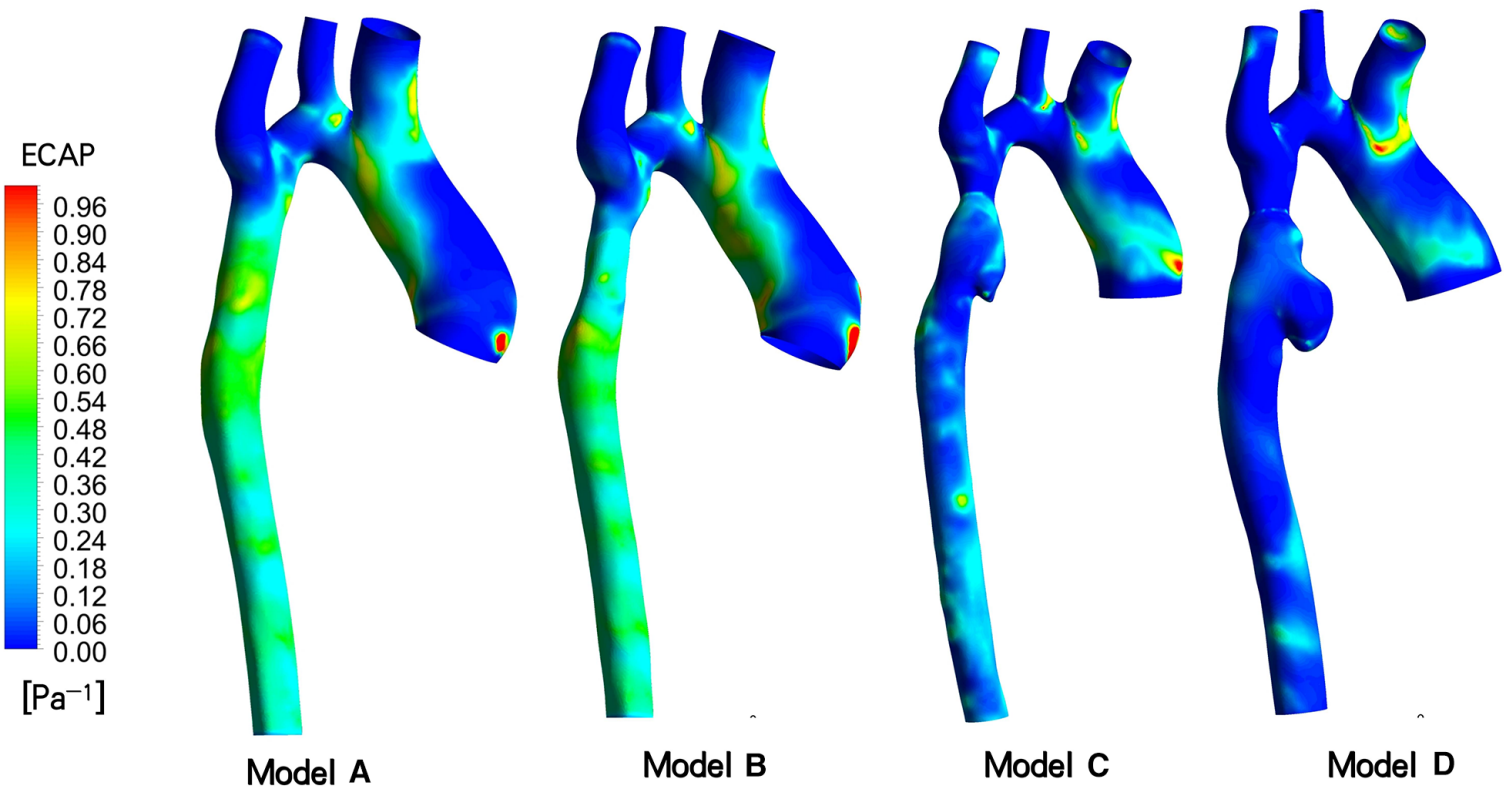
Endothelial cell activation potential (ECAP) contour models A to D indicated different follow-up time.

ECAP, which represents the ratio of two parameters, OSI and TAWSS, serves as an indicator for assessing the susceptibility to thrombosis in aneurysms (12). As depicted in Figure 6, the distribution of ECAP in the distal aneurysm region of the stent varies across the four models. Results from two postoperative follow-up visits reveal a gradual decrease in vascular wall ECAP in this area over time, with values lower than those observed during the initial intraoperative intervention and in the ideal stenosis-free model. Moreover, the ECAP value in all models is consistently below 1.4 Pa-1. Specifically, in the ideal model A, the ECAP is primarily concentrated in the range of 0.42 to 0.75 Pa^−1^, while in model B, it ranges from 0.20 to 0.80 Pa^−1^. Additionally, model C exhibits a focus on 0.05–0.35 Pa^−1^, and model D is concentrated in the range of 0.00–0.15 Pa^−1^.

## Discussion

Coarctation of the aorta (CoA) is a prevalent congenital heart condition that prompts the development of robust collateral circulation in the descending aorta to ensure adequate blood supply to the lower limbs. Unlike typical patients with constricted arches, the distal end of the stenosis did not exhibit dilation due to the collateral blood supply. Consequently, the CP stent and BIB were chosen based on the diameter at the distal end of the stenosis to address this unique characteristic.

In large arterial vessels, the normal WSS typically falls within the range of 1–5 Pa (7). When this range is exceeded, the vessel's anti-inflammatory and anti-thrombotic responses can be altered. Particularly in cases of arterial stenosis, the vessels downstream of the stenosis may facilitate aneurysm dilation due to the elevated WSS (17, 18). A comprehensive evaluation of the risk of associated complications under various CoA stent implantation strategies necessitates careful consideration of hemodynamic alterations. Given that aneurysm dilation, rupture, and thrombosis are attributed to the collective hemodynamic effects occurring throughout the cardiac cycle, it is essential to concurrently assess multiple parameters such as TAWSS, OSI, and ECAP for comprehensive risk evaluation (19–22).

In the specific case under consideration, as observed in the DSA image, the second balloon expansion resulted in the stent becoming shorter and wider, during the shortening process, it is prone to causing damage to the intima. The results presented in Table 1 and Figure 4 illustrate the evolving regional morphology and local TAWSS of the aneurysms over time. The aneurysm exhibited slight dilation in the short axis direction and significant growth in the long axis direction. This phenomenon can be attributed to the low TAWSS of the vessel wall in the long axis direction of the aneurysm at 3 days post-surgery (Model C), while the TAWSS at this location in Model D returned to the normal range at 18 months post-surgery. This discrepancy led to rapid expansion of the aneurysm in the long axis direction in the early stage, potentially resulting in relative stability in the long term. In contrast, the TAWSS of the artery wall in the short axis direction of the aneurysm only marginally increased at 18 months post-surgery, contributing to the gradual expansion of the aneurysm in the short axis direction (23–25).

Several studies have indicated a correlation between localized aneurysm dilation and the rapid growth of intraluminal thrombus within the aneurysm (26). As depicted in Figure 5, aneurysms developed in the narrow distal blood vessels following overexpansion of the CP stent, with the OSI in this area significantly lower than that of model B, which underwent incomplete initial dilation. Multiple eddy currents were observed in the aneurysm region, potentially contributing to the low OSI. Subsequent imaging revealed no thrombosis in the patient's aneurysm region. However, while numerous studies suggest a noteworthy negative correlation between intravascular OSI and thrombus aggregation in aneurysms, indicating that low OSI regions may promote thrombosis (27, 28), other studies argue that the relationship between intra-aneurysm OSI and thrombus deposition cannot be solely determined based on OSI (29). Consequently, it is challenging to employ OSI and eddy currents conjointly to evaluate aneurysm thrombosis in this case.

Considering the prevailing viewpoint that TAWSS and OSI collectively contribute to thrombosis formation, we introduce the related parameter ECAP for analysis. Generally, an ECAP value exceeding 1.4Pa-1 is indicative of a high probability of thrombosis. Simulation results for this patient indicate that the ECAP value in the distal vascular region of these stent models remains below this critical threshold, irrespective of the initial incomplete stent dilation or subsequent excessive dilation. Consequently, we posit that the risk of thrombosis in aneurysms is low. The factors contributing to rapid aneurysm dilation demonstrate limited correlation with thrombus formation.

## Study limitations

The present study utilized a simplified shell unit model and conducted a pure fluid analysis without considering the vessel wall thickness and its elastic structure beyond the point of coated stent implantation. Notably, the vessel wall typically comprises three layers - endothelium, midthelium, and ectothelium - each with unique properties. Furthermore, the patient's endothelium experienced minor rupturing during surgery, giving rise to a medically originated pseudoaneurysm with distinct vascular properties compared to other segments of the vessel wall. There is potential for predicting the growth rate and morphology of pseudoaneurysms through fluid-solid coupling (30, 31). Additionally, we implemented standardized boundary conditions to alleviate the impact of these variables, allowing for a more focused comparison of hemodynamic differences within the lesion area. However, this approach may introduce some bias in interpreting the results. Specifically, despite the thick side branch of the descending aorta distal to the stenosis of the aortic arch, which contributes to lower limb blood flow. Upon removal of these collateral vessels, blood flow and WSS in the abdominal aorta at and below this location will decrease, leading to a reduction in intracranial eddy currents to some extent. However, given the focus of this paper, the alteration in the hemodynamic environment of the thoracic aorta will not be addressed. For patients with extremely severe CoA, the blood supply to the descending aorta will no longer primarily rely on collateral vessels post-stenting. Consequently, based on the postoperative reexamination images of the patients, the collateral vessels also adaptively diminish in size, thereby reducing their original blood supply function. Hence, it is reasonable to disregard the influence of collateral vessels on the hemodynamic environment of aneurysms in the simplified model presented in this paper.

## Conclusions

Our model demonstrated that after the initial moderate stent expansion, the hemodynamic parameters of Model B were very close to those of the idealized normal Model A, suggesting a low risk of serious complications following the procedure. In contrast, when the diameter of both ends of the CP stent significantly exceeded that of the descending aorta due to overexpansion, the resulting asymmetric aneurysm exhibited low TAWSS and ECAP in the distal vascular wall. This scenario would likely promote the rapid expansion and growth of pseudoaneurysms in the early stage. Notably, the distribution range of TAWSS along the vascular wall provides important guidance for predicting the potential for aneurysm expansion.

## Data Availability

The original contributions presented in the study are included in the article/Supplementary Material, further inquiries can be directed to the corresponding author.
